# Genomic and Phylogenetic Characterization of Novel, Recombinant H5N2 Avian Influenza Virus Strains Isolated from Vaccinated Chickens with Clinical Symptoms in China

**DOI:** 10.3390/v7030887

**Published:** 2015-02-25

**Authors:** Huaiying Xu, Fang Meng, Dihai Huang, Xiaodan Sheng, Youling Wang, Wei Zhang, Weishan Chang, Leyi Wang, Zhuoming Qin

**Affiliations:** 1 Institute of Poultry Science, Shandong Academy of Agricultural Sciences, Jinan, Shandong 250100, China; E-Mails: hyingxu@163.com (H.X.); hhddhh@163.com (D.H.); sxd1985wl@163.com (X.S.); wangyouling71@163.com (Y.W.); weizhang1128@126.com (W.Z.); 2 Department of Preventive Veterinary Medicine, College of Veterinary Medicine, Shandong Agricultural University, Tai’an, Shandong 271018, China; E-Mails: mfshd@163.com (F.M.); wschang@sdau.edu.cn (W.C.); 3 Shandong Jianmu Biological Pharmaceutical Co., Ltd., Jinan, Shandong 250100, China; 4 Animal Diseases Diagnostic Laboratory, Ohio Department of Agriculture, Reynoldsburg, OH 43068, USA

**Keywords:** avian influenza virus, H5N2, recombination, genomic characterization

## Abstract

Infection of poultry with diverse lineages of H5N2 avian influenza viruses has been documented for over three decades in different parts of the world, with limited outbreaks caused by this highly pathogenic avian influenza virus. In the present study, three avian H5N2 influenza viruses, A/chicken/Shijiazhuang/1209/2013, A/chicken/Chiping/0321/2014, and A/chicken/Laiwu/0313/2014, were isolated from chickens with clinical symptoms of avian influenza. Complete genomic and phylogenetic analyses demonstrated that all three isolates are novel recombinant viruses with hemagglutinin (HA) and matrix (M) genes derived from H5N1, and remaining genes derived from H9N2-like viruses. The HA cleavage motif in all three strains (PQIEGRRRKR/GL) is characteristic of a highly pathogenic avian influenza virus strain. These results indicate the occurrence of H5N2 recombination and highlight the importance of continued surveillance of the H5N2 subtype virus and reformulation of vaccine strains.

## 1. Introduction

Infection of poultry with diverse lineages of H5N2 avian influenza viruses has been documented for over three decades [1]. One of the earliest H5N2 viruses was a low pathogenic avian influenza (LPAI) virus in chickens in Pennsylvania in April 1983. Interestingly, less than one year after that outbreak the virus was found to have mutated into a highly pathogenic avian influenza (HPAI) H5N2 strain after adaptation in birds and causing high mortality in poultry [2]. Since then, both LPAI and HPAI H5N2 strains have been identified frequently among domestic and wild bird populations worldwide [3,4,5,6,7,8,9,10,11].

HPAI H5N2 strains are generated from mutations in the hemagglutinin (HA) gene of the LPAI H5N2 virus strains. Examples of these H5N2 strains include the HPAI H5N2 virus strains in Pennsylvania (1983) and in Mexico (1994) [1,7]. In addition to genomic mutations, several lines of evidence have demonstrated that the novel HPAI H5N2 viruses may also result from reassortment [4,6,9,12]. For example, the HPAI H5N2 strain (A/chicken/Hebei/1102/2010) isolated in China in 2010 resulted from a reassortment event, with the HA being derived from the clade 7.2 (H5N1) viruses and the remaining genes being derived from the endemic H9N2 viruses [13]. To date, reassortant H5N2 viruses detected in domestic poultry belong to H5 clades 2.3.2, 2.3.4 or 7.2, and the represented reassortment events involve genes of different subtypes, including H3, H5, H6 H7, H9, and H11viruses [6,13,14,15,16,17].

Reassortment frequently occurs within a confined geographic area, such as countries or continents. However, HA and neuraminidase (NA) genes of a recombinant strain (A/Ck/Taiwan/1209/03 (H5N2)) isolated from chickens in Taiwan were found to be closely related to an American H5N2 strain and the remaining genes correlated with the Eurasian lineage [4]. Further, all genes except nonstructural (NS) of a recombinant strain (A/SWG/Nigeria/08 (H5N2)) isolated from a wild bird in Africa were found to be closely related to the Eurasian H5N2 isolates, while the NS gene is closely related to the African lineage [18]. Therefore, intercontinental exchange and dissemination events are likely to occur.

Therefore, to control avian influenza in poultry, it is critically important to track the evolution and epidemiology of H5N2 strains in the field. In the present study, we conducted genomic and phylogenetic analysis of three H5N2 influenza virus strains isolated from chickens that showed symptoms of avian influenza virus (AIV) and succumbed to infection-related death in the Hebei and Shandong province from 2013 to 2014.

## 2. Materials and Methods

### 2.1. Chicken Population

Chickens from three farms (one from Hebei and two from Shandong) were immunized with an inactivated, bivalent influenza vaccine (Re-4, H5N1, clade 7.1 and Re-6, H5N1, clade 2.3.2). Five rounds of immunization were applied to these birds at 3, 8, 16, 20, and 35 week-old ages, respectively. The birds of the three farms were 240–550 days old when ailing or dead chickens were observed.

### 2.2. Virus Detection and Isolation 

Ailing or dead chickens (210 and 60, respectively) with clinical symptoms of AIV infection were collected from one Hebei province farm and two Shandong province farms during 2013 and 2014. Liver, oviduct and lung tissue samples were collected separately from each chicken and were transported to the laboratory at 4 °C in media containing antibiotics. The tissue samples were then made into slurries, and tested by Matrix (M) gene specific RT (reverse transcription)-PCR (forward primer M-229U, 5’-TTCTAACCGAGGTCGAAAC-3’ and reverse primer M-229L, 5’-AAGCGTCTACGCTGCAGTCC-3’). The pooled samples from five positive birds of each farm were used for inoculation into the allantoic cavities of 10-day-old specific-pathogen-free chicken embryonic eggs and incubated at 37 °C for 72 h. The allantoic fluid was collected and used to determine the presence of virus with hemagglutination assay. All virus isolates were further purified with a plaque assay, sequenced and used in subsequent research.

### 2.3. RT-PCR and Sequencing

Subtyping of the HA and NA genes, and genotyping of the other six viral genes, was carried out by RT-PCR followed by sequencing. Briefly, RNA was extracted from the allantoic fluid using a Viral RNA Mini Kit (Qiagen, Hilden, Germany) and PCR amplification of the specific viral genes was carried out using a PrimeScript™ One-Step RT-PCR Kit (TaKaRa, Dalian, Japan). All viral genes were amplified with primers as described previously [19]. PCR products were purified using the QIAEX II Gel Extraction Kit (Qiagen) and cloned into the pMD18-T vector (TaKaRa). To ensure accuracy of the detected sequence, clones were sequenced using a Big Dye Terminator V.3.0 Cycle Sequencing Ready Reaction Kit (Applied Biosystems Inc., Foster City, CA, USA) for each cloned gene.

### 2.4. Genomic and Phylogenetic Analysis

**Figure 1:**
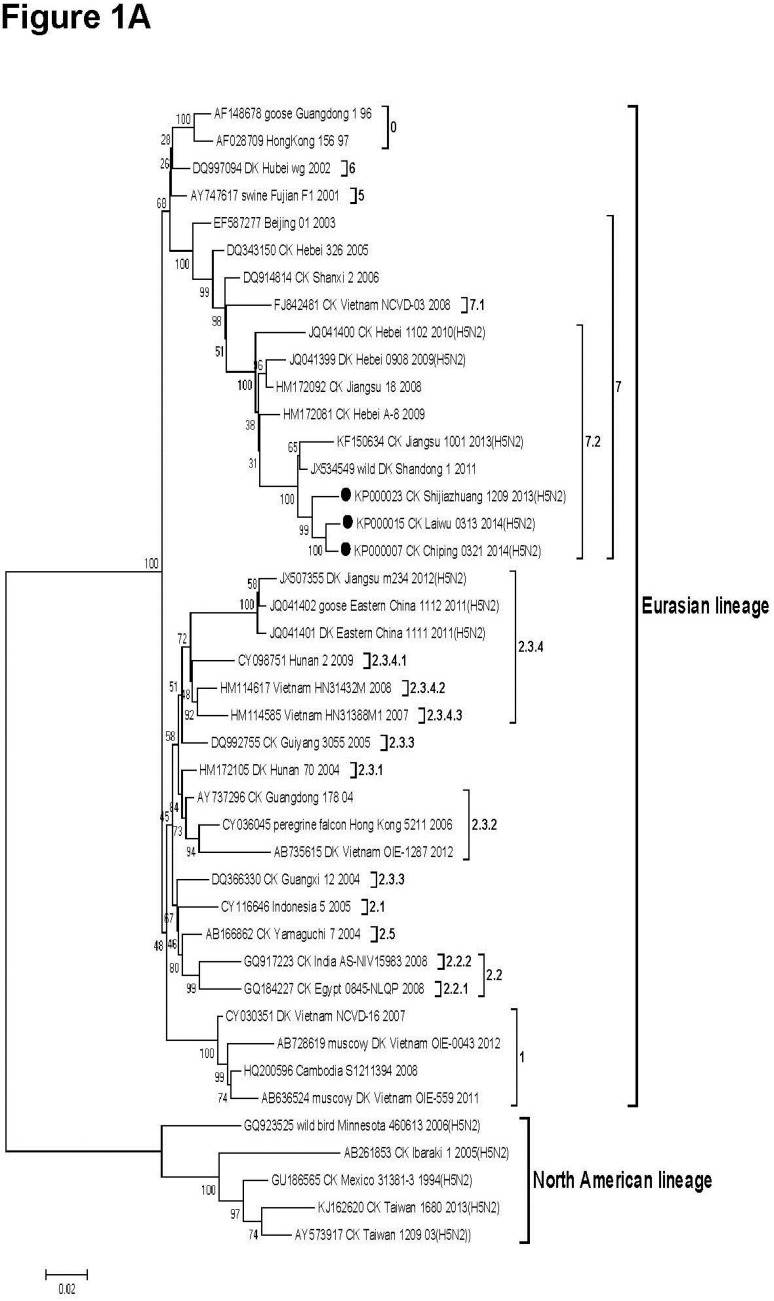

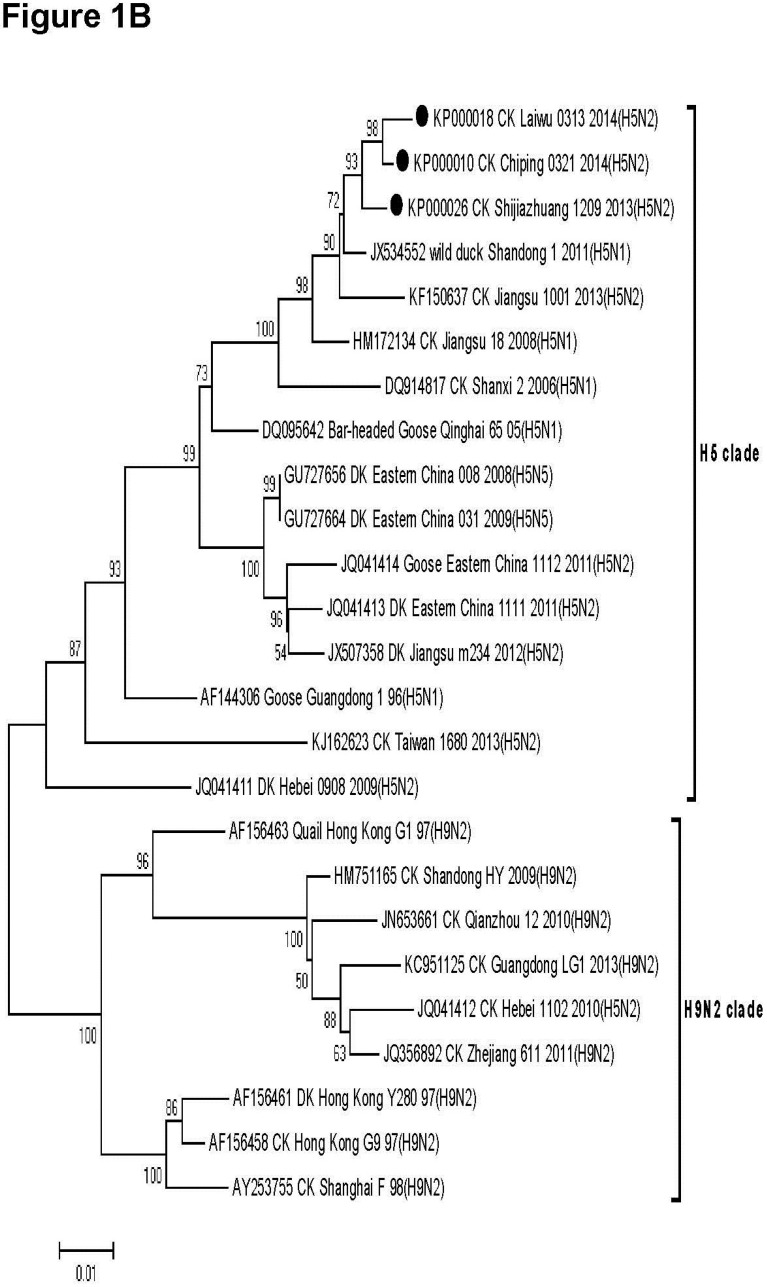

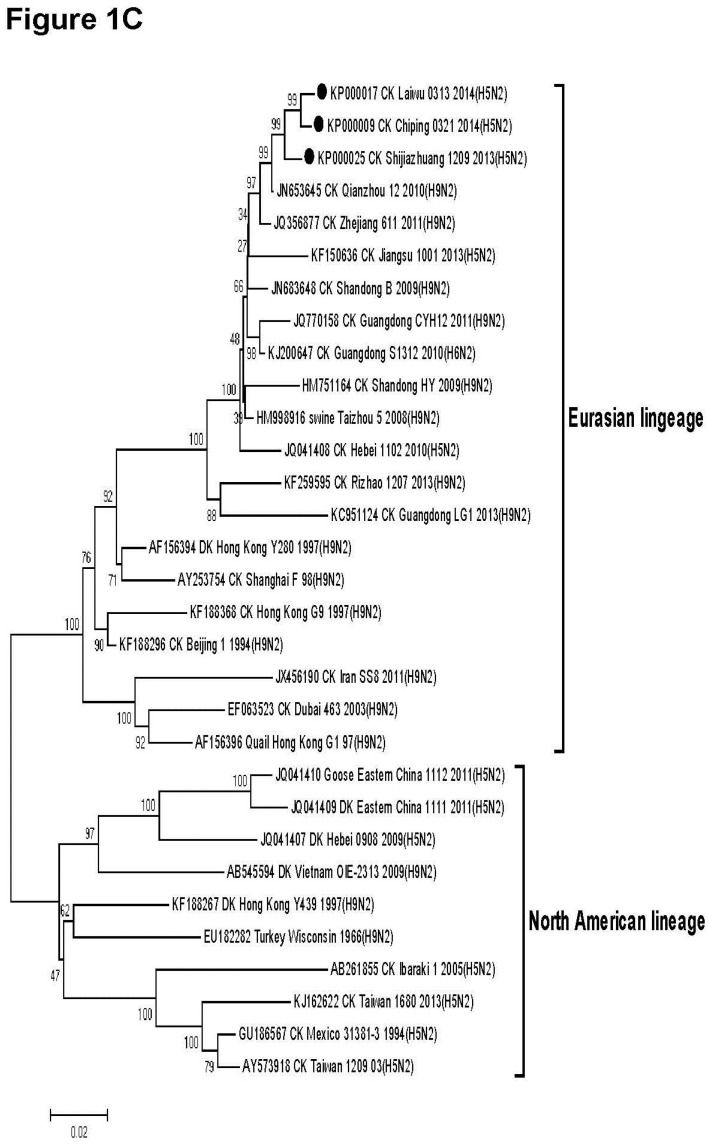
Phylogenetic trees based on the open reading frame sequences of the hemagglutinin (HA) gene (**1A**); matrix (M) gene (**1B**) and neuraminidase (NA) gene (**1C**) of H5N2 viruses in this study and those of H5N1 and H9N2 reference strains from GenBank. Viruses highlighted with a solid circle were characterized in this study. The trees were constructed using the neighbor-joining method of MEGA 5.05 with 1000 bootstrap trials performed to assign confidence to the grouping.

To explore the origin of the H5N2 virus isolates, all eight viral genes were subjected to genomic and phylogenetic analyses. Gene sequences of the AIVs reported in GenBank were selected to construct the phylogenetic trees by using the neighbor-joining method and bootstrapping with 1000 replicates in MEGA 5.05 software. H5N1, H5N2 and H9N2 classical strains of Eurasian and North American lineages, and H9N2 and H5N2 strains currently circulating in the field were selected into the phylogenetic analyses. A total of 39, 28 and 22 strains of AIVs from GenBank were used for polygenetic analyses of HA, NA, and M genes, respectively and GenBank accession numbers are listed before strain names in Figure 1.

### 2.5. Ethical Approval

This study was carried out with pre-approval obtained by the Shandong Administrative Committee for Laboratory Animals. All procedures involving the handling of animals were reviewed and approved by the Shandong IACUC (Permission number: SDXK-LD-2014-0004).

## 3. Results

### 3.1. Virus Detection and Isolation

Out of 270 birds, 252 tested positive by M gene-specific RT-PCR. Once determined positive for H5N2, all birds from these three farms were depopulated. Three H5N2 avian influenza virus strains were then isolated from chickens with clinical symptoms of AIV from each farm. The three isolates were named as A/chicken/Shijiazhuang/1209/2013(H5N2) (Ck/SJZ/1209/13), A/chicken/Chiping/0321/2014(H5N2) (Ck/CP/0321/14), and A/chicken/Laiwu/0313/2014(H5N2) (Ck/LW/0313/14). All three isolates were sequenced and their sequences have been deposited into GenBank under accession numbers KP000004 to KP000027.

### 3.2. Phylogenetic Analysis

All three H5N2 AIV isolates clustered with H5N1 subtype HA genes from clade 7.2, (Figure 1A) and shared the highest nucleotide homology with A/wild duck/Shandong/1/2011(H5N1) (97.6% to 97.7%, Table 1). The M gene from the three isolates also clustered with that of H5N1 strains and shared the highest nucleotide homology with A/wild duck/Shandong/1/2011(H5N1) (99%, Table 1, Figure 1B). In contrast, although the NA genes of the three isolates clustered in the Eurasian sublineage, the NA genes from all three isolates revealed a close relatedness (98.6% to 98.9%) to the H9N2 strain, A/chicken/Qianzhou/12/2010 (H9N2) (Figure 1C). Phylogenetic analysis revealed PB2, PB1, PA, NP and NS were also derived from an H9N2-like strain, with the closest relatedness as follows: PB2,A/chicken/Qianzhou/12/2010 (H9N2); PB1, A/chicken/Jiangsu/Q3/2010 (H9N2); PA, A/chicken/Shandong/397/2010 (H9N2); NP, A/chicken/Zhejiang/611/2011 (H9N2); NS, A/chicken/Qianzhou/12/2010 (H9N2) (Table 1). These data suggest that all three H5N2 viruses are novel recombinant isolates, having acquired HA and M genes from the H5N1 virus of A/wild duck/Shandong/1/2011(H5N1) and remaining genes from H9N2-like isolates (Figure 1, Table 1).

**Table 1:** Influenza viruses with highest nucleotide identity in each gene of CK/CP/0321/2014, CK/LW/0313/2014 and CK/SJZ/1209/2013.

Gene * Segment	CK/CP/0321/2014		CK/LW/0313/2014		CK/SJZ/1209/2013	
	Closest Virus	Nucleotide Identity, %	Closest Virus	Nucleotide Identity, %	Closest Virus	Nucleotide Identity, %
PB2	A/chicken/Qianzhou/12/2010(H9N2)	98	A/chicken/Qianzhou/12/2010(H9N2)	98	A/chicken/Qianzhou/12/2010(H9N2)	98
PB1	A/chicken/Jiangsu/Q3/2010(H9N2)	98	A/chicken/Jiangsu/Q3/2010(H9N2)	98	A/chicken/Jiangsu/Q3/2010(H9N2)	98
PA	A/chicken/Shandong/397/2010(H9N2)	99	A/chicken/Shandong/397/2010(H9N2)	99	A/chicken/Shandong/397/2010(H9N2)	99
HA	A/wild duck/Shandong/1/2011(H5N1)	98	A/wild duck/Shandong/1/2011(H5N1)	98	A/wild duck/Shandong/1/2011(H5N1)	98
NP	A/chicken/Zhejiang/611/2011(H9N2)	98	A/chicken/Zhejiang/611/2011(H9N2)	98	A/chicken/Zhejiang/611/2011(H9N2)	98
NA	A/chicken/Qianzhou/12/2010(H9N2)	99	A/chicken/Qianzhou/12/2010(H9N2)	99	A/chicken/Qianzhou/12/2010(H9N2)	99
M	A/wild duck/Shandong/1/2011(H5N1)	99	A/wild duck/Shandong/1/2011(H5N1)	99	A/wild duck/Shandong/1/2011(H5N1)	99
NS	A/chicken/Qianzhou/12/2010(H9N2)	98	A/chicken/Qianzhou/12/2010(H9N2)	98	A/chicken/Qianzhou/12/2010(H9N2)	98

* Abbreviations: PB, polybasic protein; PA, polymerase acidic protein; HA, hemagglutinin; NP, nucleocapsid protein; NA, neuraminidase; M, matrix protein; NS, nonstructural protein.

### 3.3. Molecular Characteristics

For all three of the H5N2 isolates, the HA gene was found to comprise a 1704 nucleotides open reading frame (ORF), coding for 568 amino acids, including a 16 amino acid signal peptide. Molecular analysis revealed these isolates harbor amino acid sequences characteristic of HPAI strains at the HA cleavage site (PQIEGRRRKR/GL), a determinant of high pathogenicity in birds.

It has been known that the consensus receptor binding site (RBS) for all identified HA proteins is composed of three major structural elements: a 190-helix (residues 188–194), a 220-loop (residues 221–228), and a 130-loop (residues 134–138). The conservative residues of the three H5N2 isolates within the RBS, including E190, R220, G225, Q226 and G228 (numbering according to H3), were all present, implying they retain typical avian virus-like receptor specificity (sialic acid -2,3-NeuAcGal).

## 4. Discussion

In this study, three H5N2 influenza virus strains isolated from chickens were identified as novel reassortants with a highly pathogenic viral genotype. Surprisingly, the affected birds had been vaccinated with killed influenza vaccines but still showed characteristic clinical symptoms of avian influenza and eventually died. These results are in agreement with previous work indicating that AIVs can continue genetic evolution under vaccination pressure [20]. Moreover, this study highlights the importance and necessity of periodic reformulation of the vaccine strain according to the strains circulating in the field in countries where vaccines are applied to control avian influenza. Therefore, we recommend that routine surveillance to monitor the influenza viral evolution in the field be carried out in combination with a comprehensive control program and vaccination as opposed to vaccination programs alone.

Several recombinant H5N2 AIV isolates have been reported in China and other countries. In 2012, three recombinant H5N2 viruses were identified in China: A/ chicken/Hebei/1102/2010 with its HA gene derived from clade 7.2 (H5N1) and the other seven genes from endemic H9N2 [12], A/chicken/Tibet/LZ01/2010 with four genes (PB2, HA, M, and NS) derived from H5N1 and the remaining genes from H9N2 [21], and A/duck/Jiangsu/m234/2012 with five genes (PB2, PA, HA, NP, and M) derived from H5N5, two genes (PB1 and NA) from H11N2, and the NS gene from H7N1 [6]. Another two recombinant H5N2 viruses have been reported very recently: A/chicken/Jiangsu/1001/2013 with the HA and M genes from H5N1 and the remaining genes from H9N2 [13]; A/duck/Zhejiang/6DK19/2013 with HA and NA from H5N2 and H6N2, respectively, the PB1 from H3N2, and the remaining genes from H5N1 [16]. The novel H5N2 recombinant strains identified in the present study have the HA and M genes derived from H5N1 and the remaining genes from an H9N2-like virus. It is also worth noting that recombinant H5N2 viruses could potentially contribute to the emergence of a novel H9N2 influenza virus [9]. These data together indicate that reassortment events among different influenza subtypes may contribute to the diversity of the viral gene pool thus potentially increasing the virulence and host spectrum. Taken together, these factors could significantly complicate immunization-based intervention strategies.

The amino acid sequence (RRRKR) of the influenza HA gene product at the cleavage site for all three viruses meets the molecular criterion put forth by the World Organization for Animal Health for an HPAI, or highly virulent, influenza strain. Furthermore, the intravenous pathogenicity index for a similar recombinant (A/chicken/Qingdao/1/2014(H5N2)) is 2.84 [22], South Korean swine H5N2 isolates have been shown to be derived from avian influenza viruses of a Eurasian lineage [23] and H5N2 virus antibodies have been detected in humans [24]. Therefore, there is increasing concern about the zoonotic potential of novel H5N2 recombinant strains. All amino acids relevant to receptor binding in the three isolates were identical to those of Gs/Gd/1/96. This strain binds to 2,3-NeuAcGal linkages of avian cellular receptors, indicating that the three recombinant H5N2 viruses have the potential to bind to avian cellular receptors. The possibility that these strains could potentially bind to human cellular receptors cannot be ruled out at this time.

## 5. Conclusions

Overall, we have identified three H5N2 influenza strains isolated from chickens in northern China that represent reassortant products between an H5N1 strain and several H9N2 strains. Results from this study highlight and reinforce the importance of vaccine strain reformulation and continued monitoring of AIVs both in domestic and wild birds.
